# The effect of an intensive care unit staffing model on tidal volume in patients with acute lung injury

**DOI:** 10.1186/cc7105

**Published:** 2008-11-03

**Authors:** Colin R Cooke, Timothy R Watkins, Jeremy M Kahn, Miriam M Treggiari, Ellen Caldwell, Leonard D Hudson, Gordon D Rubenfeld

**Affiliations:** 1 Division of Pulmonary & Critical Care Medicine, University of Washington School of Medicine, Harborview Medical Center, 325 9th Avenue, Box 359762, Seattle, Washington, 98104, USA; 2 Division of Pulmonary, Allergy and Critical Care, Leonard Davis Institute for Health Economics and the Center for Clinical Epidemiology and Biostatistics, University of Pennsylvania School of Medicine, University of Pennsylvania Medical Center, Blockley Hall, Room 723, 423 Guardian Drive, Philadelphia, PA 19104, USA; 3 Department of Anesthesiology, University of Washington School of Medicine, Harborview Medical Center, 325 9th Avenue, Box 359724, Seattle, Washington, 98104, USA; 4 Interdepartmental Division of Critical Care, University of Toronto, Sunnybrook Health Sciences Centre, 2075 Bayview Ave, Room D503, Toronto, Ontario, M4N 3M5, Canada

## Abstract

**Introduction:**

Little is known about the mechanisms through which intensivist physician staffing influences patient outcomes. We aimed to assess the effect of closed-model intensive care on evidence-based ventilatory practice in patients with acute lung injury (ALI).

**Methods:**

We conducted a secondary analysis of a prospective population-based cohort of 759 patients with ALI who were alive and ventilated on day three of ALI, and were cared for in 23 intensive care units (ICUs) in King County, Washington.

**Results:**

We compared day three tidal volume (V_T_) in open versus closed ICUs adjusting for potential patient and ICU confounders. In 13 closed model ICUs, 429 (63%) patients were cared for. Adjusted mean V_T _(mL/Kg predicted body weight (PBW) or measured body weight if height not recorded) for patients in closed ICUs was 1.40 mL/Kg PBW (95% confidence interval (CI) = 0.57 to 2.24 mL/Kg PBW) lower than patients in open model ICUs. Patients in closed ICUs were more likely (odds ratio (OR) = 2.23, 95% CI = 1.09 to 4.56) to receive lower V_T _(≤ 6.5 mL/Kg PBW) and were less likely (OR = 0.30, 95% CI = 0.17 to 0.55) to receive a potentially injurious V_T _(≥ 12 mL/Kg PBW) compared with patients cared for in open ICUs, independent of other covariates. The effect of closed ICUs on hospital mortality was not changed after accounting for delivered V_T_.

**Conclusions:**

Patients with ALI cared for in closed model ICUs are more likely to receive lower V_T _and less likely to receive higher V_T_, but there were no other differences in measured processes of care. Moreover, the difference in delivered V_T _did not completely account for the improved mortality observed in closed model ICUs.

## Introduction

Over the past decade there has been a growing body of literature demonstrating an association between high-intensity physician staffing in the intensive care unit (ICU) and improved patient outcomes [1-7], although this association is not without controversy [8]. In 2001 the Society of Critical Care Medicine published the recommendations of two task forces convened to determine the 'best' ICU practice model and to define the role and practice of an intensivist. Based on available evidence, the report recommended that care in the ICU "...should be led by a full-time critical care-trained physician who is available in a timely fashion to the ICU 24 hours per day" [9]. The National Quality Forum Safe Practices Recommendations, the Centers for Medicare and Medicaid Services pay for performance proposals and The Leapfrog Group make similar recommendations [10-12].

Despite widespread recommendations for ICUs to adopt high-intensity physician staffing, little is known about the mechanisms through which physician staffing influence patient outcomes. Many investigators speculate that greater intensivist presence in the ICU improves the rapidity of diagnostic and therapeutic interventions for critical patients, improves the triage and timely discharge of ICU patients and improves coordination of communication with other ICU providers [13-15]. One compelling hypothesis is that patients whose care involves an intensivist may receive more evidence-based therapies known to improve outcomes [15,16].

We recently determined that high-intensity physician staffing is associated with decreased mortality in a population-based cohort of patients with acute lung injury (ALI) [17]. One possible explanation for this finding is that closed model ICUs more strictly adhere to evidence based ALI specific care. In this study, we tried to understand the patient, hospital and provider characteristics associated with the use of lung protective ventilator settings. We hypothesised that closed model ICUs would recognise patients with ALI more frequently, deliver lower tidal volumes, measure height, weight and plateau pressure more frequently, and be more likely to deliver non-zero positive end expiratory pressure (PEEP) compared with open model ICUs.

## Materials and methods

The institutional review board at the University of Washington approved the study. Consent was waived as the collected data was made anonymous after completion of the parent study.

### Patient cohort

The King County Lung Injury Project (KCLIP) was a large, prospective, multi-centre study that measured the incidence and outcomes of ALI in King County, Washington [18]. From April 1999 to July 2000, all mechanically ventilated patients in King County, Washington, and those in neighbouring hospitals caring for King County residents were screened using a validated algorithm to identify those meeting consensus definition for ALI or acute respiratory distress syndrome (ARDS) [19]. A total of 1113 patients were enrolled in the study. Trained chart abstractors collected demographics, laboratory results, physiological data, ventilator parameters, comorbidities and ALI risk factor, provider-charted differential diagnosis using a specified protocol at the time of enrolment and, when applicable, day three post-ALI onset during the study period. Waived consent was granted by the institutional review board for each participating hospital in the parent study. Further details of the study design, data collection and data quality were previously published [18].

### ICU staffing structure and process of care

In a companion study to KCLIP we developed two questionnaires designed to obtain information about the structure, organisation, interactions among providers and process of care in KCLIP ICUs. The surveys targeted both the nurse manager responsible for each ICU represented in the KCLIP hospitals and the medical director or the attending physician with a daily presence in each KCLIP ICU. Surveys were distributed between June and December 2000; however, respondents were asked to assess practices during the cohort study periods. Further details on the survey tool were previously published [17].

### Variable definitions

Our main exposure of interest was the ICU staffing model. We defined closed staffing model ICUs as units in which patient care was directed by an ICU team or units where consultation from a board-certified intensivist was mandatory for all patients admitted to the ICU [4]. Other ICU staffing models were considered open. We defined academic ICUs as ICUs where medical trainees participate in the care of critically ill patients. We determined the volume of mechanically ventilated patients cared for in each KCLIP hospital during the study period using the Washington State Comprehensive Hospital Abstract Reporting System. Presence of ALI in the provider differential diagnosis was abstracted from the patient's chart and hospital discharge summary at the time of the original study.

Our two primary outcomes of interest were the delivered tidal volume (V_T_) on day three of ALI and the proportion of patients receiving lower and higher V_T _on day three of ALI. We defined lower V_T _as less than or equal to 6.5 mL/Kg of predicted body weight or measured body weight if height not measured (PBW). Higher V_T _was defined as 12 mL/Kg PBW or above. A sensitivity analysis was conducted by broadening our definition of protective V_T _to 8 mL/Kg PBW or less, selected to include the 95% confidence interval (CI) for V_T _reported in the 6 mL/Kg PBW arm of the acute respiratory distress syndrome Network (ARDSNet) low V_T _study [20].

Secondary outcomes included: documentation of the diagnosis of ALI or one of several synonyms in the medical record; measurement of patient height; measurement of plateau pressure during the first three days of ALI; and the use of higher levels of PEEP on day three of ALI. For the analysis of V_T _delivery, we limited the cohort to patients who were alive and ventilated on day three of ALI. Day three of mechanical ventilation was selected to allow time for recognition of ALI and implementation of lung protective ventilation.

### Statistical analysis

We calculated bivariate associations for patient and ICU level characteristics between open and closed model ICUs using student's t-test, Wilcoxon rank-sum and chi-square test as appropriate. We assessed the independent effect of staffing model on process of care using logistic regression for dichotomous V_T _and linear regression for continuous V_T_. Generalised estimating equations with exchangeable correlation were used to account for the correlation between patients in the same ICU [21,22]. We used the jackknife to calculate standard errors for the regression of V_T _versus ICU model [23].

We considered age, gender, Acute Physiology and Chronic Health Evaluation (APACHE) III score at ALI onset, ALI risk factor (sepsis or other), academic status, operative status of the patient and chest X-ray severity at ALI onset (>50% alveolar opacity in three or more quadrants versus otherwise) to potentially confound the relationship between ICU staffing model and delivered V_T_. We also sequentially added additional covariates in a sensitivity analysis to determine the influence of other variables on the staffing/V_T _relationship. One hospital was an outlier with respect to the volume of mechanically ventilated patients (1720 ventilated patients/year, n = 230) and participated in the ARDSnet low tidal volume study. This hospital was excluded as part of the sensitivity analysis. We also evaluated our secondary outcomes in multivariable regression when significant (p < 0.25) on bivariate analysis.

To determine if the reduction in mortality associated with a closed ICU, previously reported in the larger parent cohort [17], was confounded by V_T_, we noted the change in the odds ratio (OR) of death for ICU model when V_T _was added to a regression of mortality on ICU model.

All reported p values are two sided assuming p = 0.05 is statistically significant. Analyses were conducted using Stata V9.2 (Statacorp, College Station, TX).

## Results

**Figure 1:**
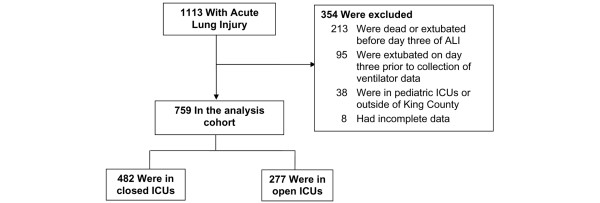
**Cohort flowchart**. ALI = acute lung injury; ICU = intensive care unit.

During the KCLIP study period, 1113 patients with ALI were identified. We excluded 354 patients from our analysis because of death or extubation before day three of ALI, hospitalisation in a paediatric hospital or hospitalisation outside of King County (Figure 1). The 759 remaining patients were cared for in 23 ICUs of which 10 followed an open staffing model and 13 followed a closed staffing model. The mean (standard deviation (SD)) board certified intensivist weekday coverage of open ICUs was 6.8 (6.3) hours compared with 7.3 (3.9) hours in closed ICUs (p = 0.84). There were no differences between ICUs with regards to presence of pharmacist on rounds (89% versus 91%, p = 0.71) or in use of a protocol for mechanical ventilation (89% versus 73%, p = 0.38) for open compared with closed, respectively. Closed ICUs were more likely to be academic and reside in hospitals caring for large volumes of mechanically ventilated patients, but these differences did not reach statistical significance (Table 1).

**Table 1:** Characteristics of intensive care units (ICUs) and patients by ICU staffing model.

Characteristic*	**ICU model**		**p value**
		
	**Open**	**Closed**	
**ICUs (N)**			
Total number	10	13	
Patients/ICU, mean (range)	28 (5 to 82)	37 (1 to 97)	0.42
Academic	5	9	0.42
Hospital volume of mechanically ventilated patients†			0.07
Median	336	578	
Interquartile range	265 to 500	421 to 1720	
**Patients**			
Total number	277	482	
Age, years	66 (15)	57 (18)	<0.001
Female	43%	37%	0.09
Race			0.55
White	73%	71%	0.03
Black	5%	10%	
Asian	4%	5%	
Hispanic	1%	2%	
Other or unknown	17%	12%	
APACHE III score	90 (29)	87 (30)	0.34
PaO_2 _to FiO_2 _ratio	147 (63)	149 (67)	0.63
Day 1 tidal volume (cc/Kg PBW)||	11.2 (2.5)	10.0 (2.2)	<0.001
ALI risk factor			<0.001
Sepsis	83%	68%	
Trauma	0%	11%	
Other	17%	21%	
>50% alveolar opacity in three or more quadrants on chest X-ray	43%	32%	<0.01
Postoperative admission	22%	22%	0.86
Pulmonary consultant involved in care	76%	81%	0.08

* represents mean (standard deviation) unless otherwise noted. Percentages may not add to 100% due to rounding.

† excludes patients (n = 52) cared for in the federal hospital in King County

||Data missing for 12 (4%) of patients in open ICUs and 32 (7%) of patients in closed ICUs.

ALI = acute lung injury; APACHE = Acute Physiology Assessment and Chronic Health Evaluation; FiO_2 _= fraction of inspired oxygen; PaO_2 _= partial pressure of arterial oxygen; PBW = predicted body weight or measured body weight if height not recorded.

Day one V_T _in patients in open ICUs was 11.2 cc/Kg PBW compared with 10.0 cc/Kg PBW in closed ICUs (p < 0.001). Bivariate associations for the primary and secondary outcomes and ICU model are shown in Table 2. A higher proportion of patients in closed ICUs received lower V_T _regardless of the definition of lower V_T _(≤ 6.5 (11% versus 5%, p = 0.004) or < 8 mL/Kg PBW (28% versus 16%, p < 0.001)). Higher V_T _(≥ 12 mL/Kg PBW) were less frequently applied in patients cared for in closed ICUs (10% versus 31%, p < 0.001). There were no differences between ICU types in the proportion of patients with 'ALI' or 'ALI or pulmonary oedema' charted in the provider's differential diagnosis. Plateau pressure was more often measured by day three of ALI in patients cared for in closed model ICUs (80% versus 69%, p < 0.001). There were no differences in PEEP at day three of ALI between closed and open model ICUs.

**Table 2:** Primary and secondary outcomes by intensive care unit (ICU) staffing model.

Patient outcome*	**ICU model**		**p value**
		
	**Open (n = 277)**	**Closed (n = 482)**	
Day 3 tidal volume			
Mean (mL/Kg)	10.8 (2.9)	9.3 (2.3)	< 0.001
≤ 6.5 mL/kg (%)	5	11	0.004
< 8 mL/kg (%)	16	28	< 0.001
≥ 12 mL/kg (%)	31	10	< 0.001
Presence in charted differential diagnosis (%)			
Acute lung injury	34	37	0.47
Acute lung injury or oedema	46	47	0.83
Height measured (%)	81	80	0.90
Weight measured (%)	99	99	1.00
Plateau pressure measured by day 3 (%)	69	80	< 0.001
Day 3 plateau pressure, mmHg‡	27 (8)	25 (8)	< 0.001
Day 3 PEEP, median (IQR) †	5 (5 to 8)	5 (5 to 10)	0.22

*represents mean (standard deviation) unless otherwise noted

† IQR = interquartile range; PEEP = positive end expiratory pressure (missing in n = 81).

‡ Data available for 167 patients in open and 301 patients in closed ICUs

**Figure 2:**
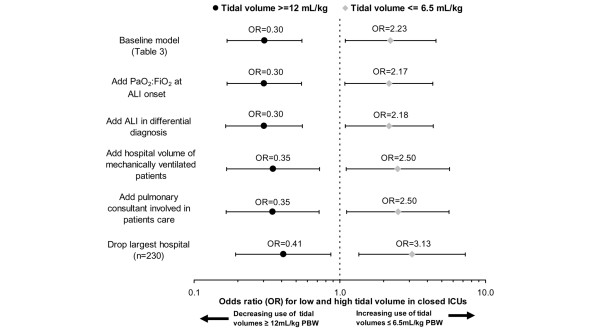
**Sensitivity analysis for regression model of the effect of closed ICU on the odds of delivery of higher (≥ 12 mL/Kg predicted body weight (PBW), left panel) and lower (≤ 6.5 mL/Kg PBW, right panel) tidal volumes**. Each covariate was sequentially added to the baseline model indicated in Table 3. Each point represents the odds ration (OR; closed versus open) after the addition of the covariate listed. Left of the dotted line indicates lower likelihood of the outcome for closed versus open ICU. Right of the dotted line indicates greater likelihood of the outcome.

On adjusted analysis, the mean V_T _for patients cared for in closed model ICUs was 1.40 mL/Kg PBW (95% CI = 0.57 to 2.24 mL/Kg PBW) lower than patients in open model ICUs. On dichotomising V_T _into 6.5 mL/Kg PBW or less, patients in closed ICUs were more likely (OR = 2.23, 95% CI = 1.09 to 4.56) to receive lower V_T _compared with patients cared for in open ICUs, independent of other covariates (Table 3). This relationship persisted when expanding the definition of lower V_T _s to include V_T _less than 8 mL/Kg PBW. Moreover, patients in closed ICUs were also less likely to receive higher (≥ 12 mL/kg PBW) V_T _(OR = 0.30, 95% CI = 0.17 to 0.55) compared with patients in open ICUs. The effect of closed model ICU on delivered V_T _was robust to changes in the included covariates in the regression model and changes in the study cohort (Figure 2). Results were similar on deletion of the outlier hospital.

**Table 3:** Adjusted odds ratio (OR) of lower and high day 3 delivered tidal volume for closed compared with open model intensive acre units (ICUs)

Covariate*	**Adjusted OR for outcome (day 3 tidal volume†)**					
	**≤ 6.5 mL/Kg**		**< 8 mL/Kg**		**≥ 12 mL/Kg**	
			
	OR	95% CI	OR	95% CI	OR	95% CI
ICU model						
Closed	2.23	1.09–4.56	2.09	1.19–3.65	0.30	0.17–0.55
Open	1.00	Referent	1.00	Referent	1.00	Referent

* adjusted for age, gender, Acute Physiology Assessment and Chronic Health Evaluation (APACHE) III at acute lung injury (ALI) onset, ALI risk factor, operative status of patient, chest x-ray severity at ALI onset, academic status

† Kg predicted body weight or measured body weight if height not measured

Adjusting for day three V_T _in multiple regression analysis had no influence on the effect of ICU model on hospital mortality. The OR for hospital death in closed versus open ICUs on bivariate analysis was 0.73 (95% CI = 0.52 to 1.02) which was similar to the OR of 0.68 reported in the larger parent cohort without adjusting for V_T_[17]. After adjusting for day three V_T_, the OR for hospital death was 0.74 (95% CI = 0.52 to 1.04). In multiple regression, there were no differences in the presence of other ALI quality indicators for closed ICUs compared with open ICUs. The likelihood of having plateau pressure measured by day three for patients in closed ICUs versus open ICUs was an OR of 0.91 (95% CI = 0.17 to 4.76). Day three PEEP level was no different for patients in closed versus open ICUs (mean difference = 0.3 mmHg, 95% CI = -1.0 to 1.0 mmHg).

## Discussion

We observed that ALI patients cared for in closed model ICUs were more than twice as likely to receive V_T _of 6.5 mL/Kg PBW or less and were less than half as likely to receive potentially injurious V_T _(≥ 12 mL/Kg PBW). These findings were independent of severity of illness, other patient-related and ICU-related factors, and were not associated with documentation of a diagnosis of ALI by the attending physicians. In addition, the beneficial effect of a closed ICU model on patient mortality was not explained by the differences in V_T_. Other features of evidence-based ventilatory care in ALI such as measuring height, weight or plateau pressure, administration of PEEP greater than zero and provider recognition of ALI did not differ between closed or open model ICUs.

It is important to note the difference between this analysis and those our group has previously reported [17]. In the current analysis, we limited the cohort, originally described by Treggiari colleagues, to patients alive and ventilated on day three of ALI in order to allow for the recognition of ALI and implementation of low V_T_. As a result of the reduced number of patients and ICUs included in the analysis, some of our reported ICU characteristics differ from those previously reported.

The established association between the closed ICU model and improved patient outcomes has led to widespread calls by public and private stakeholders to implement the closed model in ICUs [9-12]. Despite promulgation of these recommendations many ICUs have not adopted a closed model [24]. Confronting the shortage of intensivists [25] and the high costs associated with ICU restructuring [26], ICUs are in need of strategies to improve patient outcomes within the constraints of limited increase in intensivist staffing. To date, however, there are few studies describing the mechanisms through which high intensity physician staffing in an ICU improve patient outcomes. Establishing that the mechanisms by which specific ICU staffing models exert their apparent benefits could provide implementable, non-staff-dependent ways to improve patient outcomes during a period of predicted intensivist shortage.

We were surprised to find that the association between closed ICU models and decreased ALI mortality was not attenuated after accounting for V_T_. This finding suggests that V_T _may not be the primary method by which closed model ICUs reduce mortality in ALI patients. There are several possible explanations for this result. Intensivist staffing may increase use of evidence-based practices not captured in this cohort. Two studies indicate that increased intensivist staffing was associated with increased compliance with a number of evidence-based practices recommended in patients with ALI [16,27]. These studies corroborate our results and support the notion that greater intensivist presence results in greater compliance with evidence-based care. Intensivist staffing may not only lead to greater implementation of evidence-based practices but also to more timely patient evaluation, improved efficiency and fewer complications of ICU care [14,28-30]. Finally, our failure to note an important confounding effect of V_T _in the intensivist-mortality association may be due to unmeasured indication bias. Patients with ALI who received lower V_T _may have been more ill, particularly those with lower thoracic compliance. Compliance was not measured completely in this cohort which could have mitigated any confounding effects of V_T_.

Our results support a large body of literature that shows that measures of structure (eg, ICU organization), process (eg, use of lung protective ventilation), and outcome (eg, risk-adjusted mortality) do not necessarily correlate with quality [31,32]. These results also support the decision of bodies such as the Joint Commission of Accreditation of Healthcare Organizations to measure quality along multiple domains. Their proposed critical care performance measures include both process and outcome measures [33].

We recognise several limitations to our analysis. Our cohort was captured before publication of the ARDSNet study, which determined that pressure limited lower V_T _ventilation decreased mortality in patients with ALI [20]. Thus, standard of ventilatory care in ALI patients during the KCLIP study period was not established. Nevertheless, we believe our results can still be generalised to current practice for several reasons. First, multiple investigators and critical care societies recommended the use of lower V_T _in ALI long before results of the ARDSnet low V_T _study were published [34-37]. Second, evidence suggests that V_T _were slowly decreasing before ARDSnet [38]. Third, despite the publication of the ARDSnet low V_T _study in 2000, there is conflicting evidence about the ventilatory practice in current patients with ALI; many are still ventilated above V_T _targets recommended by current guidelines indicating the similarity between our cohort and recently published cohorts of patients with ALI [39-44]. Finally, our study did not assess the absolute rate of uptake of lower V_T _with new evidence, but the differences in practice between open and closed ICUs.

As with other observational studies, our results may be subject to bias as a result of residual confounding or misclassification. Recent literature suggests there is wide variation in organisational characteristics among ICUs reporting compliance with the high intensity physician staffing model [45]. We assigned ICU model structure based on definitions used in a recent systematic review of physician staffing patterns [4], but our assignment of ICU staffing model could have been in error. Although the patient level data was detailed, some variables that may play a role in selecting ventilator settings, for example, thoracic compliance, response to PEEP trial and computed tomography imaging, were not available in all patients for inclusion in the analysis.

## Conclusion

The improved outcomes associated with high-intensity physician staffing in the ICU are complex and likely to be multifactorial [13-16]. Our results suggest that ALI patients cared for in closed model ICUs receive better evidence-based care reflected by their lower V_T_; however, this difference does not explain the lower mortality of ALI patients cared for in closed ICUs. Additional research is needed to identify the mechanisms by which closed ICUs exert their influence on patient outcome.

## Key messages

• ALI patients cared for in closed model ICUs receive lower V_T_, independent of other patient and ICU characteristics.

• Lower V_T _delivered to patients in closed model ICUs are not responsible for the reduced hospital mortality associated with care in a closed model ICU.

• Patients cared for in closed versus open ICUs were equally likely to have their ALI recognised by providers, have plateau pressure recorded, and have their height or weight charted.

## Abbreviations

ALI: acute lung injury; APACHE: acute physiology assessment and chronic health evaluation; ARDSNet: acute respiratory distress syndrome network; CI: confidence interval; ICU: intensive care unit; KCLIP: King County Lung Injury Project; OR: odds ratio; PBW: predicted body weight; PEEP: positive end expiratory pressure; SD: standard deviation; V_T_: tidal volume.

## Competing interests

The authors declare that they have no competing interests.

## Authors' contributions

CRC conceived the study, performed the statistical analysis, interpreted the results and drafted the manuscript. TRW participated in data analysis and critical review and revision of the manuscript. JMK participated in study design and conceptualisation, interpretation of the results and helped in drafting the manuscript. MMT and EC were responsible for data acquisition, statistical analysis and critical review and revision of the manuscript. LDH participated in study conceptualisation and helped draft the manuscript. GDR participated in study design and conceptualisation, data collection, interpretation of the results and drafting the manuscript. All authors read and approved the final manuscript.
